# In Vitro and In Vivo Assessment of Pharmacokinetic Profile of Peramivir in the Context of Inhalation Therapy

**DOI:** 10.3390/ph18020181

**Published:** 2025-01-29

**Authors:** Liuhan Dong, Juanwen Hu, Qiannan Zhang, Mengmeng Yang, Wenpeng Zhang, Xiaomei Zhuang

**Affiliations:** State Key Laboratory of Toxicology and Medical Countermeasures, Beijing Institute of Pharmacology and Toxicology, Beijing 100850, China; donglh_cy@163.com (L.D.); jwenhu0905@163.com (J.H.); zqn1217yolo@163.com (Q.Z.); y11meng@163.com (M.Y.); wpzhang@bmi.ac.cn (W.Z.)

**Keywords:** peramivir, inhalation administration, pharmacokinetics, in vivo–in vitro correlation

## Abstract

Objective: The aim was to evaluate the pharmacokinetics and underlying mechanisms of peramivir, a clinically approved antiviral agent for severe influenza, subsequent to airway inhalation in rats, thereby surmounting the constraints associated with the sole currently available intravenous formulation. Methods: Pharmacokinetic and tissue distribution investigations of peramivir were carried out in rats following both intravenous and inhaled administration. In vitro cell models were verified to investigate peramivir’s transmembrane transport and cellular uptake across diverse cell systems. Results: In vivo, peramivir exhibited restricted permeability, predominantly localizing within the alveolar epithelial lining fluid and lung tissue after inhalation, accompanied by minimal systemic dissemination. In vitro, it manifested low permeability across cell models, with no participation of efflux transporters. Despite the low rate of A549 uptake, the underlying uptake transport mechanism was still revealed. Peramivir was verified as an OCTN2 substrate. A robust correlation was observed between the in vitro and in vivo findings. Conclusions: A preclinical pharmacokinetic platform applicable to inhaled medications was established. Inhalation of peramivir augments exposure at the target site while diminishing systemic exposure, presenting potential therapeutic benefits in terms of efficacy and safety and suggesting it as a favorable alternative administration pathway.

## 1. Introduction

Influenza is a severe acute respiratory viral disease that poses a substantial hazard to human health. Owing to its extreme infectiousness, elevated morbidity rate, extensive prevalence, and significant fatality rate, it is currently classified as one of the global infectious diseases that remain inadequately controlled [1]. In recent years, the prevalence of influenza has been rising annually due to climatic and environmental influences, with the virus undergoing fast mutations, thereby posing a significant global public health challenge [2]. Currently, there are three main categories of anti-influenza pharmaceuticals: neuraminidase inhibitors (NAIs), agents targeting M protein, and antiviral medications derived from traditional Chinese medicine. NAIs constitute a category of antiviral agents that obstruct influenza A and B viruses [1,3,4]. They possess broad-spectrum antiviral properties and are presently the most efficacious medications against influenza viruses due to the highly conserved neuraminidase active sites across influenza A viruses, influenza B viruses, and all influenza virus subtypes [4].

Peramivir, the first intravenous formulation for influenza A and B, possesses a long half-life, fast onset, and extended duration of activity [5,6]. Peramivir has demonstrated strong selective efficacy against the neuraminidase of influenza A and B viruses both in vitro and in vivo [7]. PHE guidelines indicate that oral oseltamivir is typically regarded as the first-line treatment for uncomplicated influenza; however, it may induce dizziness, insomnia, headache, behavioral changes, encephalitis, and other unpleasant effects, as well as having the potential for the virus to develop drug resistance over time [8]. A meta-analysis [1] indicates that intravenous peramivir is more efficacious and safer than oral oseltamivir for treating pediatric influenza. Despite peramivir not being included as a first-line treatment for influenza, perhaps due to its brief approval duration, substantial clinical evidence suggests that peramivir may be the most effective alternative for expediting the resolution of influenza symptoms [9]. Intravenous peramivir is a preferable option when patients cannot administer oral oseltamivir due to medical reasons related to influenza infection or in cases of oseltamivir resistance.

However, peramivir is only available as an intravenous formulation and is required to be administered for 5 to 10 days, preferably within the initial 2 days of influenza symptoms [10,11]. Consequently, notwithstanding the benefits of intravenous peramivir, patients may not choose it promptly upon the occurrence of the illness due to the inconvenience of its administration, which usually requires injections by a healthcare provider. Moreover, influenza viruses can be transmitted through the air, and the administration of peramivir via infusion may elevate the risk of infection for healthcare personnel during standard care procedures [12,13]. Therefore, it is crucial to develop a straightforward and secure method of administering peramivir that may be promptly utilized by patients and healthcare professionals to prevent and avoid influenza virus infection.

Peramivir exhibits extremely low oral bioavailability (merely 3%) due to its high polarity and low permeability through intestinal membranes. Oral administration of peramivir is associated with a reduction in virus titers but without significant relief of symptoms [12]. In recent years, numerous investigations have aimed to develop oral alternatives to intravenous peramivir to decrease hospital treatment costs and enhance patient adherence, although none are presently used in routine clinical applications [14]. The benefits and potential of pulmonary administration for respiratory illnesses render inhalation treatment an increasingly essential medical intervention. Inhalation enables direct drug delivery to the infection site, bypassing the challenges of systemic administration in the lungs, resulting in enhanced and prolonged local concentrations, thereby enhancing the therapeutic index and efficacy, reducing toxicity, and accelerating the onset of drug action. It presents new opportunities and challenges for pharmacological research and development, medication repositioning, and the clinical rational utilization of pharmaceuticals [15]. Hao Ding et al. [13] initially found the pharmacokinetics process of peramivir in rats after nasal atomization inhalation. However, what are the systemic and local exposure characteristics and the specific mechanisms following respiratory tract administration? Additionally, is there a correlation between its absorption, distribution, metabolism, and excretion (ADME) characteristics in vitro and in vivo? The existence of a pharmacokinetic benefit of the inhaled medication compared to intravenous therapy remains to be investigated and clarified.

Based on the clinical application value and the existing research gap regarding the inhaled peramivir product, we have conducted a comprehensive and systematic investigation into the preclinical pharmacokinetic characteristics of peramivir when inhaled by airway. The systemic and local exposure features following peramivir administration were thoroughly clarified using in vitro and in vivo systems. Moreover, a comparison has been made between the variations in tissue distribution following tracheal and intravenous injection. It is expected to precisely evaluate the advantages or disadvantages of the development and clinical application of peramivir inhalation formulations. Simultaneously, it is expected to provide valuable insights and a guiding direction for the progress and utilization of novel antiviral agents.

## 2. Results

### 2.1. Pharmacokinetics Study

**Figure 1:**
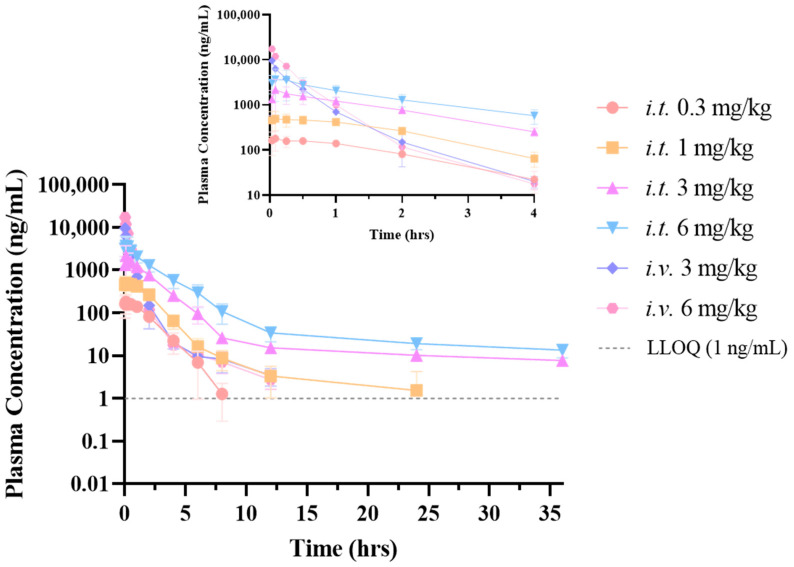
Drug concentration–time curves of peramivir in rat plasma following different administration routes and dosages. Some data are not visible due to being below the concentration of 1 ng/mL (BLQ).

The results of in vivo pharmacokinetic (PK) studies performed on healthy rats are displayed in Table 1 and Figure 1. Following intravenous treatment, the plasma concentration of peramivir in rats declined swiftly and was distributed within the initial 4 h, succeeded by a gradual elimination of peramivir over 12 h. Following inhalation, the plasma concentration peaked approximately 30 min later and subsequently declined gradually. Non-compartmental analysis (statistical moments) was employed to comprehensively characterize the pharmacokinetics of peramivir in rat plasma. Following intravenous administration of peramivir in healthy rats, the clearance rate was inferior to the hepatic blood flow (approximately 3.31 L/h/kg). The observed volume of distribution in vivo indicates that the medication does not preferentially disperse in tissues. Following intratracheal administration of varying doses of peramivir in rats, both the AUC and C_max_ exhibited a linear correlation with the dose, indicating linear pharmacokinetics within the dose range of 0.3 mg/kg to 6 mg/kg. The inhaled administration via the airway of peramivir results in higher exposure compared to intravenous injection at equivalent doses, with the mean residence time (MRT) increasing nearly tenfold, likely due to the gradual absorption of peramivir into the bloodstream via the lungs and its low systemic clearance.

### 2.2. Tissue Distribution Study

**Figure 2:**
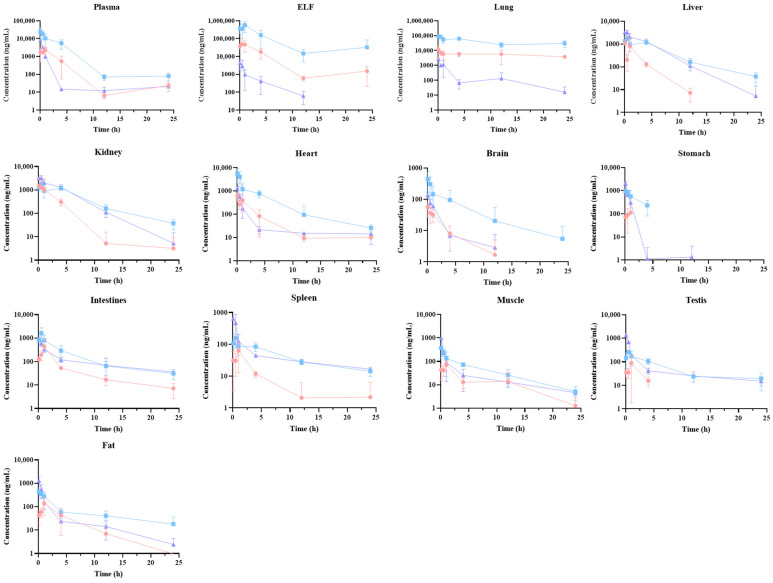
Drug concentration–time curves of peramivir in various tissues of rats following different administration routes and dosages. (red circle—*i.t.* 0.3 mg/kg, blue square—*i.t.* 3 mg/kg, purple triangle—*i.v.* 3 mg/kg, mean ± SD, n = 6).

**Figure 3:**
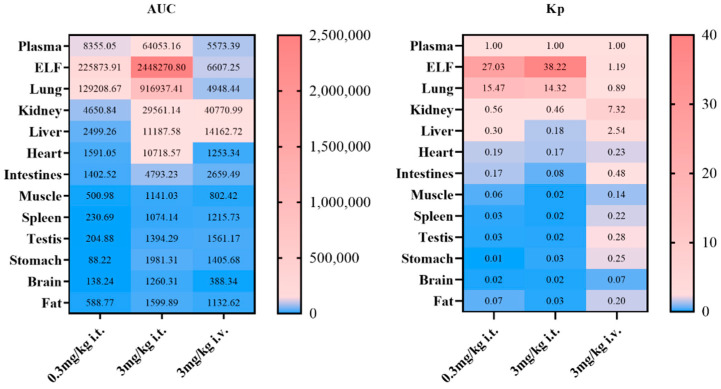
The exposure and K_p_ values of peramivir in different tissues of rats following different administration routes and dosages. (Mean ± SD, n = 6).

Following a single administration of 0.3 mg/kg and 3 mg/kg of peramivir via inhalation, as well as 3 mg/kg via intravenous injection, the concentration and tissue exposure of the drug are illustrated in Figure 2 and Figure 3. Following intravenous treatment, peramivir had modest tissue distribution, with all tissues showing lower exposure (tissue–plasma partition coefficient, K_p_ < 1) compared to plasma, except for the renal and alveolar epithelial lining, which exhibited higher exposure than plasma. This aligns with the limited permeability findings of peramivir in vitro, and the comparatively large renal exposure may be attributed to the majority being eliminated in urine in its original form. Following inhalation, peramivir exhibited predominant distribution in the lungs, particularly within the alveolar epithelial lining fluid, with exposure exceeding 370 times that of an equivalent *i.v.* dose, while distribution in other organs was comparatively little. Alongside showcasing remarkable target retention of peramivir, we noted heightened cardiac exposure via inhalation compared to intravenous treatment of the identical dose. This may result from the physiological mechanism of drug inhalation and delivery from the airway epithelium into lung tissue, subsequently entering the pulmonary vein, immediately reaching the left atrium, and ultimately circulating through the cardiovascular system.

### 2.3. Functional Validation in In Vitro Cell Models

The lung serves as a pathway for both local and systemic drug delivery, and the transporters present in the lung have an impact on drug availability and absorption. Thus, understanding the expression and function of relevant transporters in in vitro models and subsequently comparing them with in vivo levels is crucial for identifying dependable models and guaranteeing accurate extrapolations between in vitro and in vivo investigations.

The mRNA expressions of the tight junction proteins ZO-1 and occludin, efflux transporters P-glycoprotein (P-gp) and breast cancer resistance protein (BCRP), and uptake transporters, the organic cation transporters (OCTs/OCTNs), of the cell models were evaluated using q-PCR, and the results are displayed in Table S1. The integrity of the barriers was examined in various transmembrane transport models using the low-permeability medication atenolol and the high-permeability drug metoprolol. The transporter function was examined by transport assays utilizing transporter-positive substrates and inhibitors. The results are presented in Figure S1 and Table 2.

In the Calu-3 cell model, the barrier function was maintained and operating optimally. There were significant differences in the apparent permeability coefficient (P_app_) and the efflux ratio (ER values) between the groups with and without digoxin inhibitors. Digoxin, a prototypical substrate of P-glycoprotein, exhibited marked transport polarity in the Calu-3 cell model. In the inhibitor group, the ER value was approximately 1, indicating the loss of drug transport directionality, thus confirming the substantial presence of P-gp in the Calu-3 cell model. There was no significant change in the P_app(A→B)_ value and ER value between the groups with and without the BCRP inhibitor, and only the P_app(B→A)_ value was significantly different, indicating that the BCRP efflux protein was minimally expressed or not expressed in the model.

The expression levels of P-gp and BCRP in the NCI-H441 cell model and NCI-H441/dTHP co-culture model were indirectly assessed using an inhibitor approach. In the groups utilizing digoxin and its inhibitor to examine the expression of P-gp protein in the model, the group without the inhibitor exhibited no significant transport polarity in the NCI-H441 cell model. Simultaneously, the P_app_ value and ER value of the group with inhibitor did not change considerably, and the drug had no transport direction, which demonstrated that P-gp protein did not exist in the NCI-H441 cell model. There were substantial variations in P_app(B→A)_ and ER between the groups with and without the inhibitor of topotecan. Topotecan, as a typical substrate of BCRP, displayed considerable transport polarity in the NCI-H441 cell model, whereas the ER value of the inhibitor group was about 1, and the directionality of drug transport disappeared, which demonstrated the existence of BCRP in the NCI-H441 cell model.

The investigation of the functional expression of transporters in the A549 model utilized uptake data of typical substrates from various transporters by A549 within 5 min under different conditions at 4 °C and 37 °C, with or without inhibitors, as illustrated in Figure S2. The results indicated a significant difference in the cell absorption rate of the probe substrate between the inhibitor and non-inhibitor groups, suggesting the involvement of the efflux transporters P-gp and BCRP, along with the uptake transporters OCT/OCTN.

### 2.4. Acquisition of Pulmonary Absorption and Permeability Parameters

**Figure 4:**
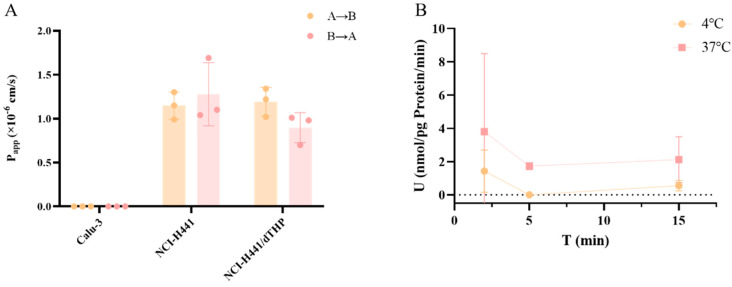
Transport assessment of peramivir in different cell models (**A**) and uptake in A549 cells (**B**) (*n* = 3).

To elucidate the penetration and absorption of peramivir through airway administration in the proximal airway and lung, the present study was carried out using an air–liquid culture model of Calu-3 cells, an air–liquid culture model of NCI-H441 cells, a co-culture model of NCI-H441/dTHP cells, and an uptake model of A549 cells. In conjunction with the outcomes of multiple in vitro studies (Figure 4 and Table 2), the absorption characteristics of drugs within the complex lung system were determined.

Currently, it is widely accepted that the P_app_ value can reflect the complexity of drug absorption. When P_app_ < 1.0 × 10^−6^ cm·s^−1^, the drug is regarded as having poor absorption, with an absorption rate ranging from 0% to 20%. A P_app_ value ranging from 1.0 × 10^−6^ to 10 × 10^−6^ cm·s^−1^ exhibits a moderate absorption rate of 20% to 70%. When P_app_ > 10 × 10^−6^ cm·s^−1^, it shows a good absorption, and the absorption rate is 70–100%. The experimental findings indicated that peramivir demonstrated limited permeability across all three in vitro transport systems. Simultaneously, there was no significant difference in permeability between the NCI-H441 cell monoculture and the NCI-H441/dTHP cell co-culture models, suggesting that pulmonary macrophages did not facilitate peramivir uptake. The results of A549 uptake experiments showed that the uptake rate of peramivir in the A549 cell model was low and that the uptake was minimal, which may be connected to its low permeability. The absorption rate at 37 °C was higher than that at 4 °C, indicating the potential involvement of transporters in the uptake process.

### 2.5. Role of Drug Transporters on Peramivir Uptake

To ascertain the potential involvement of common transporters in the ADME process of peramivir in the lung, experiments were conducted utilizing cells that overexpress the transporters. The permeability of peramivir in the barrier model was markedly low, aligning with the air–liquid barrier transport assay in the lung and a prior investigation in Caco-2 cells [12]. However, based on the barrier data of LLC-PK1-MDR1 cells overexpressing P-gp protein, there is no significant difference in the P_app(B→A)_ value compared with those obtained in LLC-PK1-MOCK cells (Table 2), demonstrating that peramivir is not a substrate of the P-gp transporter.

**Figure 5:**
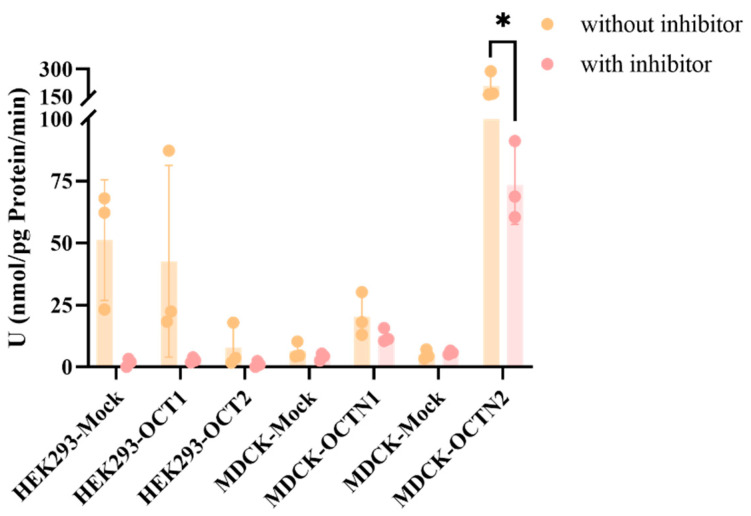
Uptake rates of peramivir in different cell models (n = 3). The substrate concentration of peramivir was 50 μM. The inhibitor of the OCT1/2 transporter was verapamil (200 μM). The inhibitor of the OCTN1 transporter was quinidine (500 μM). The inhibitor of the OCTN2 transporter was L-carnitine (200 μM). (* *p* < 0.05).

In a series of uptake studies (Figure 5) conducted in HEK293 cells engineered to overexpress the organic cation transporters OCT1 and OCT2, the URs for both transporters were found to be below the threshold of 1. This observation suggests that peramivir does not act as a substrate for OCT1 and OCT2. Despite this, the cellular uptake of peramivir was observed to be completely abrogated by the presence of the inhibitor, with an IR value exceeding 98.51%. In MDCK cells that overexpress the OCTN1 and OCTN2 transporters, the transporter-mediated uptake ratio (UR value) for the OCTN1 transporter was determined to be 3.19, which is above the threshold of 2. However, the inhibition ratio (IR value) for the inhibitor group was recorded at 40.22%, falling short of the 50% mark, and thus, it does not conclusively establish peramivir as a substrate for OCTN1. The UR value for the OCTN2 transporter was significantly higher at 41.62, surpassing the threshold of 2. Moreover, the IR value for the group treated with an inhibitor was 65.69% (>50%), thereby confirming peramivir as a substrate for the OCTN2 transporter.

## 3. Discussion

Peramivir has been approved for the treatment of influenza A and B in several countries. It demonstrates a strong affinity for neuraminidase and possesses a lower maximal inhibitory concentration (MIC) compared to other NAIs. According to prior research regarding the physicochemical properties and metabolic characteristics of peramivir [12,14,16], it is categorized as a BCS-III (high solubility/low permeability) and BDDCS-III (high solubility/low metabolism) drug [17]. The high polarity and diminished oral bioavailability resulting from the carboxyl and guanidine groups in the structure restrict the clinical application of peramivir. Given the numerous advantages of inhaled therapy, this study aims to develop an in vitro and in vivo model system for the thorough assessment of the ADME properties of inhaled drugs. Moreover, it endeavors to clarify the characteristics and mechanisms of systemic and local exposure to peramivir following inhalation by utilizing this system, thereby laying a theoretical foundation for future drug development and application.

Although many diseases can be treated by inhalation because of the advantages of pulmonary administration, inhalants are predominantly used for the treatment of COPD and asthma in the world [18]. Currently, the pharmacokinetic study and assessment of inhaled drugs lack systematic rigor and precision. This results in an unclear understanding of drug disposition at the target site, the absence of a definite dose–response relationship, and difficulties in pharmacokinetics/pharmacodynamics (PK/PD) studies. These issues hinder the provision of accurate guidance for clinical drug application and significantly impede the progress and utilization of inhaled medications [15,19,20]. The complex anatomy and physiology of the lung make the efficient administration and scientific evaluation of inhaled medications extremely challenging. Drug distribution through the respiratory tract has a specific ADME process. Additionally, the quantitative analysis of pulmonary drug pharmacokinetics is complicated by factors such as drug transport, metabolism, protein binding, and pulmonary redistribution after absorption [21]. Therefore, it is necessary to establish a reliable ADME evaluation system for drugs applicable to the lung.

Given the high heterogeneity of the lung, we attempted to integrate multiple cell models to thoroughly examine the drug absorption properties of lung tissue. The air–liquid culture paradigm of Calu-3 cells serves as a principal cellular model for investigating tracheobronchial epithelial permeability in vitro [19]. This cell line is one of the few respiratory cell lines capable of forming tight junctions in vitro and exhibits properties of differentiated, functional human proximal airway epithelial cells. This enables it to be utilized in lung research to mimic the airway epithelial barrier [22,23]. After cultivation, Calu-3 cells can undergo ciliogenesis, establish a polarized pseudostratified barrier, and express relevant transporters and metabolic enzymes. Simultaneously, they possess mucus secretion capabilities and may express cystic fibrosis transmembrane conductance regulator along with different ion channels, which is consistent with the characteristics of normal human tracheobronchiolar gland serous cells [23,24,25]. A549 cells are the predominant model for distal lung tissue and alveolar epithelial cells. They exhibit characteristics typical of alveolar type II (AT2) cells, such as expression of complement factors, synthesis of surfactant with intracellular lamellar bodies, and microvillous structures on the apical surface [26,27,28]. Consequently, they are frequently used to investigate the pulmonary pharmacology and toxicological properties of drugs. NCI-H441 cells demonstrated traits akin to Clara cells and type 2 alveolar epithelial cells in an air–liquid culture environment. Moreover, their trans-epithelial electrical resistance (TEER) values and transporter expression levels closely resemble those of primary cultured human alveolar epithelial cells [29]. NCI-H441 cells make up for the deficiency of the A549 cell line in forming tightly packed monolayers of polarized cells. Hence, this cell line can serve as a principal model for investigating the distal airway and alveolar epithelial barrier [30,31]. The lung contains a large number of macrophages, and their potential influence on drug metabolism and ADME properties has gained significant attention. In recent years, some in vitro models have utilized differentiated THP-1 cells for co-culture with other cells in related studies [32,33,34,35]. Previously, Ana Costa et al. [36] established and characterized a modified air–blood barrier 3D co-culture comprising epithelial NCI-H441 cells, endothelial HPMEC-ST1.6R cells, and differentiated THP1 cells, assessing the potential of nanoparticles to traverse the air–blood barrier in both healthy and proinflammatory conditions. The co-culture model developed in this study demonstrated the benefits of three-dimensional culture. Additionally, the combination of macrophage-like cells and endothelial cells with epithelial cells also exhibited a higher sensitivity in triggering a putative proinflammatory response compared to epithelial monocultures.

The inhaled route provides an important way to administer antiviral compounds that are effective against influenza. Antiviral medications such as zanamivir and laninamivir octanoate have already been approved for the treatment of influenza virus infection via inhalation [37]. Concerning zanamivir, its absolute bioavailability is notably low when administered orally, at approximately 2%. When inhaled orally, this figure rises to a range of 4% to 17%, with the drug being excreted and cleared unchanged by the kidneys. In contrast, laninamivir, which is administered as the approved prodrug laninamivir octanoate, exhibits a prolonged retention time in the lungs following pulmonary delivery and a plasma half-life that is considerably longer than that observed after intravenous administration. Generally, the pharmacokinetic profiles of these two approved dry powder inhalants are comparable to those of atomized peramivir in this study. However, due to their distinct structures and physicochemical properties, there are still notable variations in both pharmacokinetics and efficacy among the three inhalants. The research conducted by Hao Ding et al. [13] provided the first PK study of peramivir administration via trans-nasal aerosol inhalation. However, in vivo studies using the nasal inhalation exposure system are unable to accurately quantify the amount of drug that actually enters the lungs. Moreover, in vivo exposure is significantly influenced by the physiological state of the animal as well as the external environment. In contrast, the use of a small-animal nebulized lung delivery device enables precise control over the delivered dose.

Peramivir exhibits a distinct exposure profile following tracheal delivery compared to intravenous treatment. At an equivalent dosage, the plasma concentration of peramivir following inhalation is higher than that observed after intravenous treatment. To begin with, it is worth noting that both routes of administration utilized solution formulations, with the solvent being normal saline. Proceeding to our analysis, we have observed that peramivir demonstrates a “flip-flop-like” kinetic pattern when administered via inhalation, upon comparing the pharmacokinetic differences between respiratory and intravenous administration. This behavior may be attributed to peramivir’s minimal penetration into the airways and lungs, which consequently leads to a slower rate of absorption into the bloodstream. The drug initially localizes within the pulmonary tissue before entering the systemic circulation for eventual elimination. Theoretically, the interaction between the compound and the metabolic enzyme adheres to the principles outlined by the Michaelis–Menten equation. It is evident that at low substrate drug concentrations, the metabolic rate is significantly lower compared to rates observed near the maximal metabolic velocity (km). Moreover, the extensive surface area of the lung, the ample absorption sites available, and the prolonged duration of absorption also serve as critical factors contributing to the flip-flop effect. The sustained release of peramivir from the lung parenchyma reduces its systemic clearance, which may well be the primary explanation for the observed bioavailability exceeding 100%. The tissue distribution investigation of the two administration modes indicated that the exposure of the drug in the elimination organs such as the liver and kidneys after inhalation administration is lower than that after intravenous administration, which also gave an indirect explanation for this phenomenon. Concurrently, following intravenous administration, the drug is predominantly distributed in the kidneys and liver. The pulmonary exposure (including lung tissue and epithelial lining fluid) is comparable to that in plasma, although distribution in other tissues is minimal. Upon inhalation, the medication predominantly accumulates in the lungs, spreads to lung tissue via the alveolar epithelial lining fluid, and then gradually enters the bloodstream. As a result, this significantly reduces its exposure to non-target tissues. Of note, intratracheally administering peramivir may increase its concentration in the brain. However, after reviewing the literature, it did not have any potential side effects on the brain/nerve site.

Studies have showed that the IC_95_ of peramivir against various subtypes of influenza viruses ranged from 4.64 to 22.42 nM (1.31 ng/mL to 6.33 ng/mL) [6]. In this study, it was found that the lung exposure levels in rats via both routes significantly surpassed their pharmacodynamic concentrations. Moreover, at equivalent doses, inhalation led to greater lung exposure than intravenous injection. The findings suggested that administering peramivir by inhalation could significantly decrease the required dosage. The nearly flat drug–time curve of peramivir in the lungs post-inhalation indicated a consistent exposure level, reinforcing the notion of a “local” action in the lungs and enhancing its extended antiviral efficacy. Therefore, peramivir administration by the inhaled route has the potential to minimize the frequency of administration.

An in vitro study [12] of peramivir under the oral route has shown that the P_app_ values of peramivir (10 μM) were 3.29 ± 0.739 × 10^−7^ cm·s^−1^ in the Caco-2 cell model. Its limited permeability is assumed to be the main reason for its low oral bioavailability. The rationale for the distinct pharmacokinetic process following peramivir inhalation and the applicability of the in vitro model to elucidate the mechanism have emerged as significant subjects for our subsequent research. To achieve this, we have constructed an in vitro model system that can characterize the absorption characteristics of different areas of the lung. A comprehensive assessment of the permeability and absorption properties of peramivir in vitro indicated that its permeability in the proximal airway region was extremely low. In the distal airway and alveolar region, peramivir still showed low permeability, but the permeability coefficient was higher than that in the proximal airway. Additionally, alveolar macrophages had almost no impact on the permeability characteristics of peramivir in a healthy state. Meanwhile, the uptake assay in A549 cells demonstrated that peramivir undergoes passive diffusion and a certain degree of active transport in the distal airways and alveoli, albeit at a slow rate. Therefore, it is plausible to infer that peramivir predominantly enters the body via penetration through the parvobronchioles and alveoli following administration through the respiratory system.

Previous studies conducted by our group indicate that the LLC-PK1 and LLC-PK1-MDR1 cell models serve as gold-standard systems for assessing whether drugs are potential substrates and inhibitors of P-gp transporters, as well as in vitro models for the expedited evaluation of drug permeability across the blood–brain barrier. Peramivir demonstrated very low permeability in this model and did not exhibit the features of a P-gp transporter substrate. This indicates that physicochemical properties, rather than efflux transporters, are the crucial factors restricting its membrane penetration rate. This is consistent with the findings regarding peramivir in the Calu-3 cell culture exhibiting high P-glycoprotein expression and the observed limited cerebral exposure in vivo. Based on the presence of some of the major uptake transporters in the lung, we tested whether peramivir was the substrate of the corresponding uptake transporters using the corresponding overexpressed cell lines. Meanwhile, the uptake of peramivir by OCT2 was also investigated based on its high fractionation in the kidneys in vivo. Due to the poor permeability of peramivir, when 10 μM was selected as the substrate concentration, the concentration of peramivir was below the detection limit (2 nM). Therefore, we increased the substrate concentration to 50 μM, although this is not a typical practice in general uptake experiments. In the context of HEK293 cells overexpressing OCT1 and OCT2, no discernible role of the overexpressed transporters in cellular uptake was detected. Nonetheless, a pronounced inhibition of the cellular uptake rate was observed upon treatment with the inhibitor verapamil. This inhibitory effect may be attributed to verapamil’s broad-spectrum action, which affects not only OCT1 and OCT2 but also other transporters present in HEK293 cells. Regarding the OCTN1 transporter, despite evidence suggesting its involvement in peramivir uptake, as indicated by a UR value greater than 2, the addition of an inhibitor resulted in only a modest inhibition of the uptake rate (IR < 50%), implying that peramivir is unlikely to be a substrate for OCTN1. In contrast, the OCTN2 transporter demonstrated a significant contribution to the cellular uptake of peramivir, which was notably reduced by the presence of an inhibitor, as evidenced by an IR value exceeding 50%. This observation suggests that peramivir is indeed a substrate for OCTN2, although it is not completely inhibited. Unfortunately, we did not investigate the uptake of OCT3 and anion transporters such as OATP2A1 and OATP2B1 in the lung due to the unavailability of corresponding overexpression cell lines.

## 4. Materials and Methods

### 4.1. Chemicals and Reagents

Peramivir was obtained from Macklin Biochem Technology Co., Ltd. (Shanghai, China). Propranolol was purchased from Selleck Chemicals (Houston, TX, USA). The HPLC-grade acetonitrile (ACN) and methanol (MeOH) were purchased from Thermo Fisher Scientific (Waltham, MA, USA). The HPLC-grade formic acid was bought from J&K Scientific (Beijing, China). Purified water was sourced from Wahaha Group Co., Ltd. (Hangzhou, China). The urea nitrogen content detection kit was purchased from Boxbio Co., Ltd. (Beijing, China). The Pierce BCA protein assay kit was purchased from Thermo Fisher Scientific (Waltham, MA, USA). Hank’s buffer and PBS buffer were purchased from Solarbio Science & Technology Co., Ltd. (Beijing, China). DMSO (purity > 99.7%) was purchased from Innochem (Beijing, China). Tariquidar (TAR) was purchased from Shanghai Yuanye Bio-Technology Co., Ltd. (Shanghai, China). Ko143 was purchased from ChemCruz Biochemicals (Huissen, The Netherlands). Atenolol, metoprolol, verapamil, topotecan, and digoxin were purchased from Selleck Chemicals (Houston, TX, USA). Ergothioneine and L-carnitine were purchased from Macklin Biochem Technology Co., Ltd. (Shanghai, China). Quinidine was purchased from TRC (Toronto, ON, Canada). Mildronate was purchased from Shenyang Tianbang Pharmaceutical Co., Ltd. (Shenyang, China). Dexamethasone and PMA were purchased from MedChemExpress (Monmouth Junction, NJ, USA).

### 4.2. Animals

Healthy Sprague–Dawley rats (200–230 g) were purchased from Beijing Vital River Laboratory Animal Technology Co., Ltd.(Beijing, China). The animals were housed in a clean facility with a 12 h light/dark cycle and fed chow and water ad libitum at 22 ± 1 °C and 50% relative humidity. All the experiments were conducted at the Beijing Center for Drug Safety Evaluation after obtaining ethical approval (IACUC-DWZX-2023-P713) from the Institutional Animal Care and Use Committee of the Centre, following the guidelines of the Association for Assessment and Accreditation of Laboratory Animal Care International (AAALAC).

### 4.3. Cell Culture

Calu-3 cells and A549 cells were obtained from the National Biomedical Laboratory Cell Repository (Beijing, China). NCI-H441 cells and THP-1 cells were purchased from Shanghai Zhong Qiao Xin Zhou Biotechnology Co., Ltd. (Shanghai, China). LLC-PK1 cells with stable expression of human MDR1 and mock-transfected with an empty vector (LLC-PK1-MOCK) were generously provided by Prof. Qingcheng Mao from the University of Washington. The Hek293 cell lines expressing human OCT1 (Hek293-SLC22A1), OCT2 (Hek293-SLC22A2), and mock-transfected with an empty vector (Hek293-MOCK) were supplied by GenoMembrane (Yokohama, Japan). The MDCK cell lines expressing human OCTN1 (MDCK-SLC22A1) and OCTN2 (MDCK-SLC22A2) and mock-transfected with an empty vector (MDCK-MOCK) were generously provided by Prof. Huidi Jiang from Zhejiang University. All cells were genotyped by STR, matched exactly with the ATCC database, and tested negative for mycoplasma. The number of cell generations used in the experiment was within 30 generations.

### 4.4. LC-MS/MS Method Development

All samples of cell lysate, plasma, tissue homogenate, and other biological matrices were precipitated with acetonitrile (containing IS, propranolol, 5 ng/mL) followed by quantitation against the corresponding standard curve prepared in the blank biomatrix. The concentration of peramivir was determined simultaneously by an LC-MS/MS method with an AB SCIEX API 5000 triple quadrupole mass spectrometer (AB SCIEX, Framingham, MA, USA) connected to an LC-20AD HPLC system (Shimadzu, Kyoto, Japan). Chromatographic separation was achieved in a C18 column (3.0 mm × 50 mm, 2.6 μm, Phenomenex) using a mobile phase consisting of water containing 0.1% formic acid (A) and acetonitrile containing 0.1% formic acid (B). A binary gradient elution procedure was used to separate the components as follows: 0.0~0.3 min B 5%, 0.3~1.0 min B 5~60%, 1.0~2.0 min B 60~95%, 2.0~2.2 min B 95%, 2.2~2.21 min B 95~5%, and 2.21~3.0 min B 5%. The volume of each injection was 3.0 μL, and the flow rate was 0.5 mL/min. The analytes and IS were detected by multiple reaction monitoring (MRM) in the positive ion mode (Figure S3).

### 4.5. In Vivo Studies

#### 4.5.1. Pharmacokinetics Study

Pharmacokinetic studies were performed on rats to examine the effects of two distinct routes of administration: intravenous (*i.v.*) and tracheal nebulization (*i.t.*). The two administration routes were established to provide different doses, with six SD rats randomly assigned and weighed for each route.

The intravenous group consisted of rats that were randomly allocated into two subgroups, receiving dosages of 3 mg/kg and 6 mg/kg, respectively. The *i.t.* group consisted of rats that were randomly allocated into four groups receiving doses of 0.3 mg/kg, 1 mg/kg, 3 mg/kg, and 6 mg/kg. Prior to and at 0.033, 0.083, 0.25, 0.5, 1, 2, 4, 6, 8, 12, and 24 h post-administration, about 200 μL of blood was obtained via jugular vein catheterization and transferred to an EP tube containing heparin. Following centrifugation for 10 min at 5000× *g*, the plasma was isolated and preserved in a refrigerator at −40 °C until analysis.

#### 4.5.2. Tissue Distribution Study

Tissue distribution characteristics were studied following different deliveries of *i.v.* 3 mg/kg, *i.t.* 0.3 mg/kg, and *i.t.* 3 mg/kg in rats. The rats were euthanized at 5 min, 30 min, 1 h, 4 h, 12 h, and 24 h after administration. Blood was collected by puncture of the fundus venous plexus, and bronchoalveolar lavage was performed to obtain bronchoalveolar lavage (BAL) fluid samples, and then the main tissues were obtained by dissection. All the tissues were thoroughly rinsed in ice physiological saline, then weighed and stored at −40 °C for analysis.

Whole blood was placed in EP tubes containing sodium heparin, and plasma was extracted by centrifugation at 4 °C, 5000× *g* for 10 min. A certain proportion of pure water was added to each tissue for grinding to obtain tissue homogenate. Drug concentrations in the samples were determined using the LC-MS/MS method and drug concentrations in the BAL were converted to drug concentrations in epithelial lining fluid (ELF) using the urea nitrogen correction method.

#### 4.5.3. Data Analysis

Noncompartmental PK parameter estimates were determined from individual concentration-time data, using Phoenix WinNonlin, version 8.1 (Certara USA, Princeton, NJ, USA). And the linear relationship between AUC_(0–t)_ and the corresponding dose was analyzed. *t* tests were performed on pharmacokinetic parameters under different dosing regimens to analyse the differences in drug exposure in rats.

The apparent volume of ELF in BAL fluid was determined by the urea dilution method described by Rennard. The estimated concentration of drugs in ELF was calculated as follows:

C_ELF_ = C_BAL_ × (U_Plasma_/U_BAL_) (1)

where C_BAL_ is the measured drug concentration in BAL fluid and U_Plasma_ and U_BAL_ are the measured concentrations of urea nitrogen in plasma and BAL fluid, respectively.

The K_p_ of each tissue was calculated as follows:

K_p_ = AUC_(0–t, tissue)_/AUC_(0–t, plasma)_ (2)

### 4.6. In Vitro Studies

#### 4.6.1. Permeability Assays Using Different Cell Models

##### Construction and Validation of the Calu-3 Cell Model

Calu-3 cells were cultured in EMEM medium (Zhongke Maichen Technology Co., Ltd., Beijing, China) supplemented with 10% fetal bovine serum (FBS, Gibco, Thermo, CA, USA), and streptomycin–penicillin (PS, 100 U/mL) at 37 °C in a humidified 5% CO_2_ incubator, and the medium was changed every other day. Once the cell state reached a stable and optimal condition, the cells were transferred to Transwell^®^ plates (Corning, USA) at a density of 5 × 10^4^ cells/well with 200 μL of cell suspension per well. Then, 850 μL of complete medium was added to the basolateral chamber. After approximately 8 days, the TEER value was measured to be about 200 Ω × cm^2^, and the medium in the apical surface of the Transwell^®^ was removed. Subsequently, an air-interfaced culture (AIC) was initiated by supplying 650 μL of culture medium to the basolateral chamber only. The culture medium was replaced every other day.

Following approximately 14 days of air–liquid interface cultivation, the TEER value was determined to be >300 Ω × cm^2^, while the permeability value of Lucifer yellow CH dipotassium salt was found to be <2 × 10^−6^ cm/s. The functional expression of this cell model was confirmed by detecting the mRNA expression of related membrane proteins and probe substrate inhibition experiments. The effective construction of the cellular barrier was acknowledged, and subsequently, the transfer experiment commenced.

##### Construction and Validation of the NCI-H441 Cell Model

NCI-H441 cells were cultured in RPMI-1640 medium (Gibco, Thermo, CA, USA) supplemented with 10% FBS and PS (100 U/mL) at 37 °C in a humidified 5% CO_2_ incubator, and the medium was changed every other day. Once the cell state reached a stable and optimal condition, the cells were transferred to Transwell^®^ plates (Corning, NY, USA) at a density of 5 × 10^4^ cells/well with 200 μL of cell suspension per well. Then, 850 μL of complete medium (containing 200 nM dexamethasone) was added to the basolateral chamber. After approximately 4 days, the TEER value was measured to be about 200 Ω × cm^2^, and the medium in the apical surface of the Transwell^®^ was removed. Subsequently, an air-interfaced culture (AIC) was initiated by supplying 650 μL of culture medium to the basolateral chamber only. The culture medium was replaced every other day.

Following approximately 7 days of air–liquid interface cultivation, the TEER value was determined to be >300 Ω × cm^2^, while the permeability value of Lucifer yellow CH dipotassium salt was found to be <2 × 10^−6^ cm/s. The functional expression of this cell model was confirmed by detecting the mRNA expression of related membrane proteins and probe substrate inhibition experiments. The effective construction of the cellular barrier was acknowledged, and subsequently, the transfer experiment commenced.

##### Construction and Validation of the NCI-H441/dTHP Co-Culture Model

THP-1 cells were cultured in RPMI-1640 medium (Gibco, Thermo, CA, USA) supplemented with 10% FBS, PS (100 U/mL), and β-Mer (0.05 mM) at 37 °C in a humidified 5% CO_2_ incubator, and the medium was changed every other day. THP-1 cells were seeded at 4 × 10^5^ cells/mL in six-well plates and induced for 48 h by adding medium containing a final concentration of 100 ng/mL PMA. The successful differentiation into M0 macrophages was verified by morphological observation and mRNA detection.

Then, the differentiated cells were seeded at 5 × 10^4^ cells/well on the surface of NCI-H441 cells 10 days after air–liquid culture and continued to co-culture for 24 h. The TEER value was determined to be >270 Ω × cm^2^, while the permeability value of Lucifer yellow CH dipotassium salt was found to be <2 × 10^−6^ cm/s. The effective construction of the cellular barrier was acknowledged, and subsequently, the transfer experiment commenced.

##### Transport Experiment

When the cell transport system was mature in culture, experiments were performed. We removed the liquid from the apical and basolateral of the Transwell, rinsed the cells three times with a buffer containing HBSS, 10 mM Hepes, and 0.1% BSA (Buffer A), and then added Buffer A (100 µL for the apical side and 600 µL for the basolateral side) for pre-incubation for 30 min at 37 °C. For the transport studies in the apical to basolateral (A-B) direction, 100 μL of pre-warmed test solution (Buffer A containing 2 µM positive control drugs or peramivir) was added to the apical side, while 600 μL of pre-warmed assay buffer, a buffer containing HBSS, 10 mM Hepes, and 1% BSA (Buffer B), was added to the basolateral compartment. When assessing permeability in basolateral to apical (B-A) direction, 100 μL of pre-warmed Buffer B and 600 μL of pre-warmed test solutions were added to the apical and basolateral compartments, respectively. The Transwells^®^ were placed in an incubator at 37 °C and 5% CO_2_ for a duration of 2 h. At the end of the incubation period, the samples in the apical and basolateral sides were collected separately and were briefly kept at a temperature of −40 °C for LC-MS/MS analysis.

#### 4.6.2. Uptake Study by A549 Cells

A549 cells were cultured in DMEM medium supplemented with 10% FBS and PS (100 U/mL) at 37 °C in a humidified 5% CO_2_ incubator. Cells were seeded on 24-well plates at a density of 3 × 10^4^ cells/well. For the uptake assay, the DMEM media were removed, and the cells were washed with pre-warmed Hank’s buffer three times (1 mL/well). The third washing buffer was retained in the wells, and the plate was placed in an incubator at 37 °C or 4 °C for 30 min prior to the initiation of the uptake experiment. Following the removal of the washing buffer, 200 μL of pre-warmed test drug solution (Hank’s buffer containing the 10 µM peramivir) was added into each well, and the plates were incubated at 37 °C or 4 °C for 2, 5, and 15 min. Then the incubation solution was removed, and the cells were washed with ice-cold transportation buffer three times (1 mL/well). A total of 0.2 mL pure water was added to each well to lyse the cells by repeated freezing and thawing (−196 °C~37 °C, three times). The cell lysate was collected and stored at −40 °C for LC-MS/MS analysis and protein measurement.

#### 4.6.3. Permeability Assays Using LLC-PK1-MOCK and LLC-PK1-MDR1 Cells

Both cells were cultured in RPIM-1640 medium supplemented with 10% FBS and PS (100 U/mL) at 37 °C in a humidified 5% CO_2_ incubator. The cells were transferred to Transwell^®^ plates (Corning, NY, USA) at a density of 2 × 10^4^ cells/well with 200 μL of cell suspension per well, while 850 μL of complete medium was added to the basolateral chamber. The culture medium was replaced every other day, and after being cultivated for about 4~7 days, the TEER value was determined to be >70 Ω × cm^2^, at which point the transfer experiment began. The experimental methodology was as previously described. The samples were collected separately and were briefly kept at a temperature of −40 °C for LC-MS/MS analysis.

#### 4.6.4. Uptake Study by Overexpression Cell Lines

All cells (HEK293-MOCK, HEK293-OCT1, HEK293-OCT2, MDCK-MOCK, MDCK-OCTN1, and MDCK-OCTN2) were cultured in DMEM medium supplemented with 10% FBS and PS (100 U/mL), at 37 °C in a humidified 5% CO_2_ incubator. Cells were seeded on 24-well plates at a density of 1 × 10^4^ cells/well. For the uptake assay, the DMEM media were removed, and the cells were washed with pre-warmed Hank’s buffer three times (1 mL/well). Subsequent addition of inhibitor-free/inhibitor-containing buffer was pre-incubated for 30 min at 37 °C prior to the initiation of the uptake experiment. After the pre-incubating buffer was removed, 200 μL of pre-warmed test drugs solution (Hank’s buffer containing 10 µM and 50 µM peramivir) or a mixture of test drugs and inhibitor was added into each well, and the plates were incubated at 37 °C for 5 min. At the end of incubation, cells were washed and lysed as described above. The cell lysate was collected and stored at −40 °C for LC-MS/MS analysis and protein measurement. The negative control (MOCK cells) and positive control were included simultaneously.

#### 4.6.5. Data Analysis and Statistical Analysis

In the bidirectional transport study, the following equations were used to calculate P_app_ values and ER values:

P_app_ = 1/(A × C_0_) × (dx/dt) (3)

ER = (P_app,BA_/P_app,AB_) × 100% (4)

where A is the surface area of the Transwell insert, C_0_ is the initial concentration of a compound applied to the donor chamber, t is incubation time, x is the amount of a compound in the receiver chamber, and dx/dt is the flux of the compound across the cell monolayer.

In the uptake study, the uptake clearance (U) of the substrate, the transporter-mediated uptake ratio (UR), and the inhibition ratio (IR) were calculated according to following equations:

U = C_lysate_/(P × T) (5)

UR = U/U_MOCK_ (6)

IR = [1 − (U_with inhibitor_ − U_mock with inhibitor_/U_without inhibitor_ − U_mock without inhibitor_)] × 100% (7)

where P represents the protein content and T is the duration of the incubation time.

Statistically significant differences between the two groups were determined by the *t*-test. Differences were considered to be significant at *p* < 0.05.

## 5. Conclusions

In conclusion, we have successfully developed a systematic preclinical pharmacokinetic study platform for inhaled medications, which has been used in an in vivo disposition and mechanism study of inhaled drugs. The combination of in vivo and in vitro experimental results demonstrated a strong association, offering valid data support for later in vitro–in vivo extrapolation (IVIVE). We effectively employed the platform to explore the systemic and local exposure features of peramivir following inhaled delivery and fundamentally, essentially elucidated the relevant process. Inhalation of peramivir substantially increases exposure of the drug to target regions of the lungs and minimizes systemic exposure. This brings substantial advantages in enhancing the efficacy and safety of pharmaceuticals and represents a promising alternative delivery approach. Concurrently, the involvement of OCTN2 transporters in the cellular uptake of peramivir was identified for the first time. Further efforts should be made toward integrative analysis of the in vitro and in vivo features using computer modeling techniques to overcome the species barrier and extend the application of inhaled drugs to clinical use in humans.

## Figures and Tables

**Table 1:** Pharmacokinetic parameters of peramivir in rat plasma following different administration routes and dosages (Mean ± SD, *n* = 6).

Parameter	Units	*i.t.* 0.3 mg/kg	*i.t.* 1 mg/kg	*i.t.* 3 mg/kg	*i.t.* 6 mg/kg	*i.v.* 3 mg/kg	*i.v.* 6 mg/kg
T_max_	h	0.37 ± 0.38	0.67 ± 0.40	0.29 ± 0.36	0.29 ± 0.39	/	/
C_max_ or C_0_	ng/mL	204.67 ± 80.82	544.17 ± 215.23	2303.33 ± 1523.96	3866.00 ± 2454.21	12,705.55 ± 1264.79	22,731.79 ± 5543.47
C_max_/D	kg × ng/mL/mg	682.22 ± 269.38	544.17 ± 215.23	767.78 ± 507.99	644.33 ± 409.04	/	/
AUC_(0–t)_	h × ng/mL	405.17 ± 56.59	1279.25 ± 93.59	4364.35 ± 583.02	8479.33 ± 883.87	3765.02 ± 247.43	6048.04 ± 635.88
AUC_(0–t)_/D	h × kg × ng/mL/mg	1350.57 ± 188.64	1279.25 ± 93.59	1454.78 ± 194.34	1413.22 ± 147.31	1255.00 ± 82.48	1008.01 ± 105.98
AUC_(0–∞)_	h × ng/mL	407.71 ± 56.59	1291.03 ± 92.89	4673.22 ± 567.78	8852.22 ± 897.97	3773.97 ± 245.49	6060.92 ± 637.85
V_z_	mL/kg	/	/	/	/	2462.65 ± 1569.16	4432.99 ± 1013.10
Cl	mL/h/kg	/	/	/	/	797.79 ± 53.18	999.26 ± 106.91
V_ss_	mL/kg	/	/	/	/	498.11 ± 58.10	472.67 ± 63.58
T_1/2_	h	1.07 ± 0.22	4.10 ± 1.69	27.20 ± 9.67	17.73 ± 2.85	2.11 ± 1.25	3.07 ± 0.61
MRT_(0–t)_	h	1.62 ± 0.31	2.08 ± 0.69	3.15 ± 0.66	3.41 ± 1.08	0.59 ± 0.06	0.47 ± 0.06
MRT_(0–∞)_	h	1.67 ± 0.33	2.32 ± 1.07	8.26 ± 4.04	5.70 ± 1.84	0.62 ± 0.06	0.51 ± 0.07

**Table 2:** Transport results of positive substrates (2 μM) and peramivir (2 μM) in different cell models (Mean ± SD, n = 3).

Model	Drug	Papp (×10−6 cm/s)		ER
		A-B	B-A	
**Calu-3**	Atenolol	0.30 ± 0.05	0.16 ± 0.04	0.30 ± 0.05
	Metoprolol	29.82 ± 2.28	21.88 ± 3.73	29.82 ± 2.28
	Digoxin	0.66 ± 0.02	25.06 ± 7.18	0.66 ± 0.02
	Peramivir	BLQ	BLQ	/
**NCI-H441**	Atenolol	1.71 ± 0.14	1.71 ± 0.12	1.00
	Metoprolol	25.61 ± 5.45	23.4 ± 2.96	0.91
	Topotecan	1.54 ± 0.12	3.47 ± 0.26	2.25
	Peramivir	1.15 ± 0.16	1.28 ± 0.36	1.11
**NCI-H441/dTHP**	Atenolol	1.56 ± 0.17	1.38 ± 0.24	0.88
	Metoprolol	27.91 ± 7.99	24.36 ± 0.22	0.90
	Topotecan	2.88 ± 0.55	6.18 ± 1.63	2.15
	Peramivir	1.19 ± 0.16	0.9 ± 0.17	0.75
**LLC-PK1**	Atenolol	BLQ	0.18 ± 0.02	/
	Metoprolol	28.49 ± 2.17	25.60 ± 3.71	0.90
	Digoxin	1.64 ± 0.27	2.22 ± 0.36	1.35
	Peramivir	BLQ	0.21 ± 0.04	/
**LLC-PK1-MDR1**	Atenolol	1.22 ± 0.11	1.55 ± 0.19	1.27
	Metoprolol	20.40 ± 1.24	23.08 ± 1.94	1.13
	Digoxin	0.74 ± 0.15	9.76 ± 0.73	13.13
	Peramivir	BLQ	0.23 ± 0.03	/

## Data Availability

The authors declare that all the data supporting the findings of this study are available within the paper.

## Supplementary Materials

The following supporting information can be downloaded at: https://www.mdpi.com/article/10.3390/ph18020181/s1. Figure S1: Transport inhibition experiments of efflux transporter-positive substrates on Calu-3 models (A) and NCI-H441 models (B); Figure S2: Uptake assay of positive substrates in A549 cells under different temperatures with or without inhibitor; Figure S3: Typical chromatograms of peramivir (150 ng/mL) and IS (5 ng/mL); Table S1: The mRNA expression of major tight junction proteins and transporters in different cell models.
