# Supplementary material for: In Vitro and In Vivo Assessment of Pharmacokinetic Profile of Peramivir in the Context of Inhalation Therapy
Source: Pharmaceuticals (Basel). 2025 Jan 29;18(2):181. doi: 10.3390/ph18020181 (PMC11858854; doi:10.3390/ph18020181)
Supplement: Supplementary file 1 [file pharmaceuticals-18-00181-s001.zip › pharmaceuticals-3401664-supplementary.pdf]

Table S1. The mRNA expression of major tight junction proteins and transporters in different cell models (n = 6).

Cell/Gene	*TJP1*	*OCLN*	*ABCB1*	*ABCG2*	*SLC22A3*	*SLC22A4*	*SLC22A5*
Calu-3	+++	+++	+++	++	/	/	/
NCI-H441	+++	+++	++	+++	++	++	++
A549	/	/	+++	+/-	++	+	++

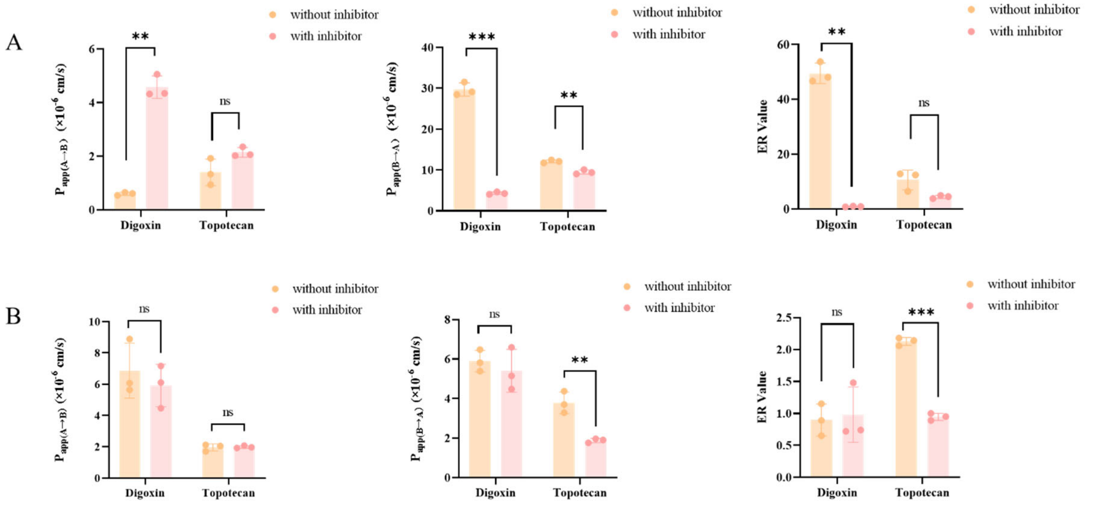

Figure S1. Transport inhibition experiments of efflux transporter-positive substrates in Calu-3 models (A) and NCI-H441 models (B) (n = 3) The substrate of the P-gp transporter is digoxin (2 μM), and the specific inhibitor used was tariquidar (5 μM). The substrate of the BCRP transporter is topotecan (2 μM), and the specific inhibitor used is ko143 (5 μM).

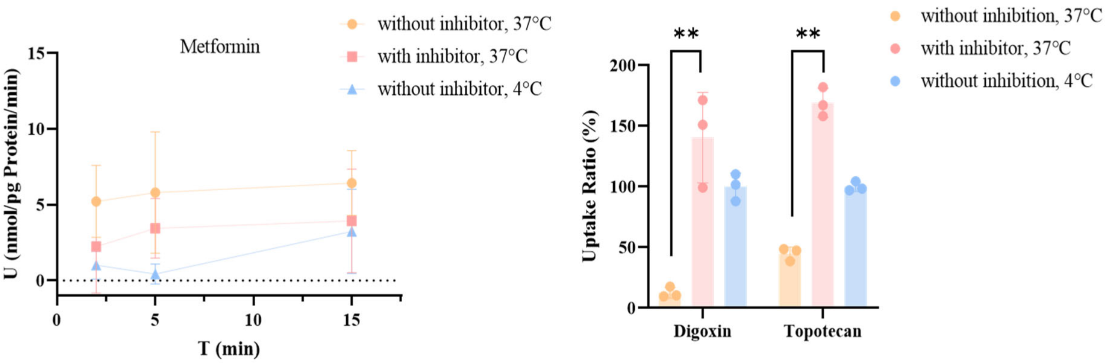

Figure S2. Uptake assay of positive substrates in A549 cells under different temperatures with or without inhibitor (n = 3) The substrate of the P-gp transporter was digoxin (5 μM), and the corresponding inhibitor was tariquidar (50 μM). The substrate of the BCRP transporter was topotecan (5 μM), and the inhibitor was ko143 (50 μM). The substrate for the OCTs/OCTNs transporter was metformin (50 μM), and the inhibitor was verapamil (200 μM).

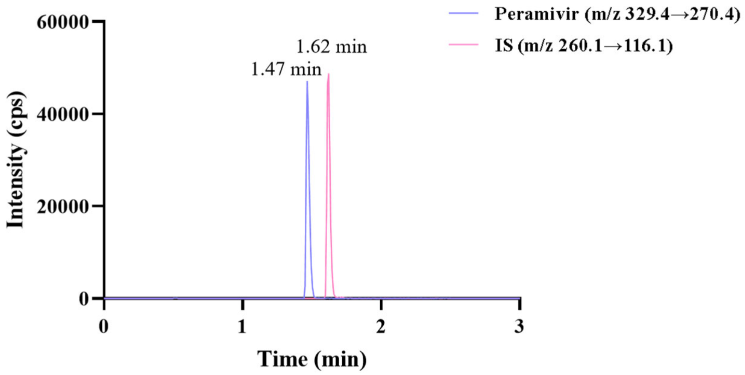

Figure S3. Typical chromatograms of peramivir (150 ng/mL) and IS (5 ng/mL).
